# Multidrug-resistant *Salmonella* Typhimurium in Four Animal Facilities

**DOI:** 10.3201/eid1108.050111

**Published:** 2005-08

**Authors:** Jennifer G. Wright, Leslie A. Tengelsen, Kirk E. Smith, Jeff B. Bender, Rodney K. Frank, John H. Grendon, Daniel H. Rice, Ann Marie B. Thiessen, Catherine Jo Gilbertson, Sumathi Sivapalasingam, Timothy J. Barrett, Thomas E. Besser, Dale D. Hancock, Frederick J. Angulo

**Affiliations:** * Centers for Disease Control and Prevention, Atlanta, Georgia, USA;; † Idaho Department of Health and Welfare, Boise, Idaho, USA;; ‡ Minnesota Department of Health, Minneapolis, Minnesota, USA;; § University of Minnesota, St. Paul, Minnesota, USA;; ¶ Washington Department of Health, Olympia, Washington, USA;; # Washington State University, Pullman, Washington, USA;; ** Chambers Creek Veterinary Hospital, Lakewood, Washington, USA;; †† The Gene Poole Memorial Cat Clinic, Bellingham, Washington, USA

**Keywords:** nosocomial infections, Salmonella infections, zoonoses, multiple drug resistance, DT 104, veterinary clinics, veterinary hospitals, salmonella enterica subsp enterica serotype Typhimurium, Salmonella serotype Typhimurium

## Abstract

In 1999 and 2000, 3 state health departments reported 4 outbreaks of gastrointestinal illness due to *Salmonella enterica* serotype Typhimurium in employees, clients, and client animals from 3 companion animal veterinary clinics and 1 animal shelter. More than 45 persons and companion animals became ill. Four independent investigations resulted in the testing of 19 human samples and >200 animal samples; 18 persons and 36 animals were culture-positive for *S*. Typhimurium. One outbreak was due to multidrug-resistant *S*. Typhimurium R-type ACKSSuT, while the other 3 were due to multidrug-resistant *S*. Typhimurium R-type ACSSuT DT104. This report documents nosocomial transmission of *S*. Typhimurium and demonstrates that companion animal facilities may serve as foci of transmission for salmonellae between animals and humans if adequate precautions are not followed.

## Abstract 2

Within each of 4 outbreaks of *S*. Typhimurium among humans and animals at companion animal care facilities, isolates were identical or nearly identical.

*Salmonella* spp. infect an estimated 1.4 million persons annually in the United States. Although most infections are self-limiting with diarrhea, vomiting, abdominal cramps, and fever, severe infections are not uncommon. Estimates suggest that ≈15,000 people are hospitalized and >500 deaths occur annually due to *Salmonella* infections (1). Food animals are the primary reservoir for human nontyphoidal *Salmonella* infections; person-to-person transmission of nontyphoidal salmonellae is uncommon in the United States. Transmission of salmonellae to humans typically occurs by ingesting meat, dairy products, and other foods contaminated by animal feces or by cross-contamination from foods contaminated with salmonellae. Zoonotic transmission of *Salmonella* spp. can also occur through direct exposure to the feces of reptiles, farm animals, pets, pet treats, and other animals (2–10).

Antimicrobial agents such as fluoroquinolones and third-generation cephalosporins (e.g., ceftriaxone) are commonly used to treat severe human *Salmonella* infections. Resistance to these and other antimicrobial drugs, as well as multidrug resistance, has increased over the last several decades (11), partly as a consequence of antimicrobial drug use in food animals (2). The use of antimicrobial agents in companion animals (e.g., dogs and cats) may also contribute to the development of antimicrobial resistance in salmonellae, but the impact of this contribution is unknown. Antimicrobial drug use in companion animals, therefore, could increase the likelihood of zoonotic transmission of multidrug-resistant salmonellae by generating drug-resistant strains as well as by making animals more susceptible to resistant infections (12).

*Salmonella* outbreaks with illness in animals and humans have been reported in both equine and companion animal veterinary facilities (13–19). These reports commonly describe poor hand washing by employees, eating in work areas, and prior antimicrobial drug therapy in humans or animals. Case reports of companion animals infected with salmonellae have included multidrug-resistant isolates (15,18–22).

In late 1999 and early 2000, 3 state health departments reported to the Centers for Disease Control and Prevention (CDC) outbreaks of gastrointestinal illness in employees and clients of 3 companion animal veterinary clinics and 1 companion animal shelter. We review these outbreaks, which demonstrate that companion animal veterinary facilities can serve as foci of transmission of salmonellae to animals and humans. Recommendations to prevent further outbreaks of salmonellosis associated with companion animal veterinary facilities were reviewed.

## Veterinary Clinic, Idaho

In September 1999, several kittens being treated for diarrhea at a veterinary clinic (clinic A) died; stool specimens were not collected. Within 2 days of the kittens' deaths, an employee who had cared for the kittens became ill with diarrhea. Days later a second employee who cared for the kittens became ill, as did other employees who had no direct contact with the kittens. Within 2 weeks, 10 (50%) of 20 employees of clinic A had experienced diarrhea and abdominal cramps. The median age of the ill persons was 31 years (range 19–44 years). The median duration of illness was 7 days (range 4–12 days). Four persons sought medical care. No one was hospitalized.

Stool specimens from 5 ill employees yielded salmonellae, which was serotyped at the Idaho Department of Health and Welfare (IDHW) as *Salmonella enterica* serotype Typhimurium. The IDHW tested all *S*. Typhimurium isolates received during the outbreak period by pulsed-field gel electrophoresis (PFGE) and for chloramphenicol resistance by using disk diffusion. Isolates were tested at CDC for resistance to 17 antimicrobial agents by using broth microdilution (Sensititre, Trek Diagnostic Systems, Cleveland, OH, USA) with Clinical and Laboratory Standards Institute (formerly NCCLS) interpretive criteria used to determine antimicrobial susceptibility (23). The 5 isolates were resistant to ampicillin, chloramphenicol, kanamycin, streptomycin, sulfamethoxazole, and tetracycline (R-type ACKSSuT); 2 isolates were additionally resistant to gentamicin, clavulanic acid, cephalothin, and ceftriaxone. Isolates were indistinguishable by PFGE. Phage testing was performed at CDC according to the methods of the Laboratory of Enteric Pathogens, Central Public Health Laboratory, Health Protection Agency, UK, and all 5 isolates reacted but did not conform (RDNC).

All ill employees had eaten meals in the clinic in the days before illness onset. A breakroom was provided for employees, yet all reported eating on work surfaces instead. The ill employees had no known common exposures outside clinic A. No additional *S*. Typhimurium isolates from Idaho were found with chloramphenicol resistance or with a related PFGE pattern during the outbreak.

## Animal Shelter, Minnesota

In Minnesota, *S*. Typhimurium isolates are routinely subtyped at the Minnesota Department of Health (MDH) Public Health Laboratory by PFGE and tested for antimicrobial susceptibility. As part of integrated human-animal surveillance, *S*. Typhimurium isolates from the Minnesota Veterinary Diagnostic Laboratory (MVDL) are routinely forwarded to MDH for PFGE subtyping and antimicrobial susceptibility testing.

**Figure 1:**
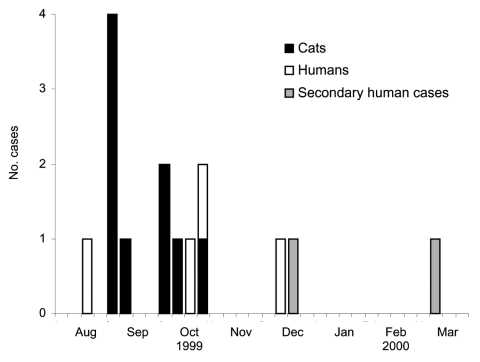
Date of death among cats and week of illness onset among human case-patients, Minnesota, 1999.

On December 2, 1999, five *S*. Typhimurium isolates from cats originating from the same county, submitted by a regional animal shelter (shelter A), were subtyped at MDH; all had the same PFGE pattern. The following day, a human *S*. Typhimurium isolate routinely submitted to MDH was determined to have the same PFGE pattern as the cat isolates. Three additional human cases were identified retrospectively with dates of illness onset from August to November; 2 reported recently adopting a kitten from shelter A. A review of recently diagnosed feline salmonellosis cases from shelter A identified 9 cases; all 9 cases were fatal and originated from multiple sources, but all ill cats were housed at shelter A. Dates of death for the kittens ranged from September through late October (Figure 1). Six of the 9 kittens had been adopted; 5 of the 6 were returned to shelter A because of illness.

**Figure 2:**
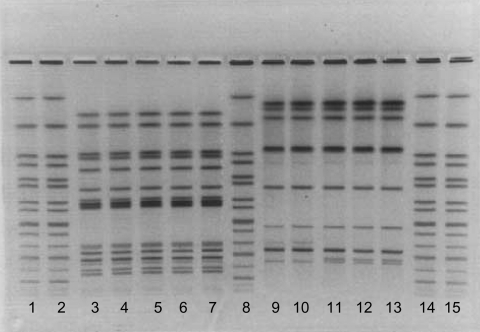
Pulsed-field gel electrophoresis patterns of human and feline isolates from Minnesota outbreak,1999. Lanes 1, 2, 8, 14, and 15 contain Xbal-digested DNA from the standard strain H9812; lanes 3, 4, 9, and 10 contain human isolates; lanes 5–7, and 11–13 contain feline isolates. Lanes 3–7 were digested with Xbal and lanes 9–13 with Blnl.

From August 1999 to March 2000, a sample of 7 human isolates and 9 feline *S*. Typhimurium isolates were identified as indistinguishable by PFGE. Figure 2 displays PFGE patterns for 4 human and 6 feline isolates. All 16 isolates were resistant to ampicillin, chloramphenicol, streptomycin, sulfamethoxazole, and tetracycline (R-type ACSSuT). Three cat and 2 human isolates were phage typed at CDC; all were definitive type 104 (DT104). Six of the 7 human case-patients with the PFGE outbreak subtype of *S*. Typhimurium had a discernable connection to shelter A; 4 had adopted kittens from shelter A during August–October 1999. Two additional patients were children who attended the same daycare center as a child who became ill 77 days after adopting 2 kittens from shelter A. This child had been treated with multiple antimicrobial drugs in the month before the onset of her salmonella gastroenteritis, including cephalexin, amoxicillin, trimethoprim-sulfamethoxazole, and ciprofloxacin. Neither of her 2 kittens developed diarrhea, but the outbreak strain of *S*. Typhimurium R-type ACSSuT DT104 was recovered from the stool of one of these cats 115 days after adoption.

The median age of the ill persons was 6 years (range 11 months–23 years). The median duration of illness was 8 days (range 5–11 days). All 7 persons sought medical care; 1 child was hospitalized for 4 nights. One of the ill persons was treated with ciprofloxacin but continued to shed *Salmonella* spp. in stool for at least 214 days after illness onset.

After the institution of enhanced infection control procedures by the shelter staff in October, no further cases of salmonellosis occurred at the facility. In December 1999, environmental samples from drains, kennels, and additional cats housed at the shelter were all negative for *Salmonella* spp.

## Clinic B, Washington

**Figure 3:**
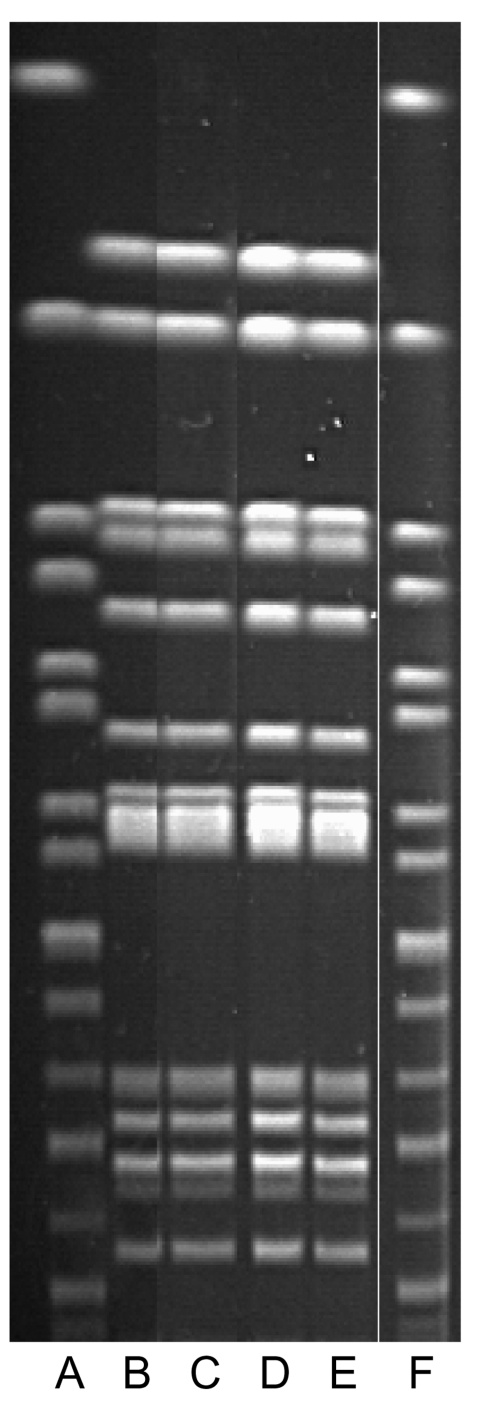
Pulsed-field gel electrophoresis patterns associated with the Washington state outbreaks, 1999 and 2000. Lanes A and F are standards; lane B is cat, clinic B; lane C is cat, clinic C; lanes D and E are human isolates.(For confidentiality reasons, Washington Department of Health did not identify which human isolates were from which outbreak.) Human and cat isolates are indistinguishable.

In late 1999, twelve cats were brought to a companion animal veterinary clinic (clinic B) with diarrhea, vomiting, anorexia, and lethargy. All 12 cats lived in different homes and all roamed outdoors. Stool specimens collected from 6 of the 12 yielded *S*. *enterica*; all isolates were serotyped at the National Veterinary Services Laboratory (NVSL) as Typhimurium. Two of 3 persons who became ill had handled ill cats. The third person had brought his cat to clinic B during the outbreak for treatment of an unrelated illness. Stool specimens from the 3 ill persons and 6 cats yielded *S*. Typhimurium, R-type ACSSuT DT 104. Isolates were indistinguishable from each other by PFGE (Figure 3).

The Field Disease Investigation Unit (FDIU) from the Washington State University College of Veterinary Medicine performed a comprehensive investigation. Medical chart reviews demonstrated that 7 (58%) of the 12 ill cats had been seen at clinic B for an unrelated reason 3–37 days (median 5 days) before gastrointestinal illness onset; 4 (57%) of these 7 cats had been treated for their original symptoms with an antimicrobial agent to which the outbreak strain of *S*. Typhimurium was later found to be resistant.

As part of this investigation, 2 controls were selected from clinic B records for each ill cat. For each case, the next cat owner listed alphabetically in clinic B's files, as well as the next cat seen at the clinic after the ill cat, was selected as a control. Distance was measured from owner's homes to the nearest creek. Affected cats owners' homes, regardless of whether the cat's infection was potentially nosocomial, were significantly closer to 1 of 2 creeks traversing the community than were control client's homes (rank sum test, p<0.01). Water samples collected from the creeks 2 months after the outbreak were negative for *S*. *enterica*.

Stool specimens for bacterial culture were collected from additional animals treated at clinic B, including 43 hospitalized cats, 37 outpatient cats, and 23 cats from households with proven infected cats. Salmonellae were isolated from 5 (12%) of 43 hospitalized cats, including the asymptomatic clinic blood donor cat and from 3 (8%) of 37 cats residing in households with infected cats; no isolates were obtained from sampled outpatient cats. The 6 original culture-positive cats were periodically sampled and shed salmonellae 3–60 days before becoming culture-negative. As part of the investigation, specimens from the remaining 6 original cats (from which no previous specimens had been cultured) were collected; these specimens were cultured and confirmed to shed the outbreak strain of *S*. Typhimurium. Fecal cultures of 26 animals from a local animal shelter were negative for *Salmonella*.

Seventy-two environmental samples were collected from clinic B, from homes of clinic B clients and employees, and from a local animal shelter. The outbreak strain of *S*. Typhimurium was isolated from the floor of the boarding area and from the floor and door handles in the clinic B isolation ward. The outbreak strain was additionally isolated from an environmental sample (a vacuum cleaner bag) from the house where 2 of the infected cats lived (24). *Salmonella* spp. were not isolated from the homes of clinic B employees, nor from the local animal shelter.

The additional *Salmonella* isolates from animals and the environmental samples were serotyped at NVSL as *S*. Typhimurium and phage typed as DT104. Agar disk-diffusion testing determined the isolates to be R-type ACSSuT. The original 6 animal isolates, the animal isolates collected during the investigation, and the human isolates were indistinguishable by PFGE.

Ten of the ill cats received empiric antimicrobial therapy for their gastrointestinal illness. When antimicrobial susceptibility results became available, the antimicrobial agent of the 7 cats still undergoing treatment was changed to enrofloxacin. Although clinical signs of the affected cats resolved promptly after initiation of 10-day courses of enrofloxacin therapy, stool cultures of specimens from 3 cats taken 14–24 days after completion of the course of enrofloxacin yielded the outbreak strain of *S*. Typhimurium.

## Clinic C, Washington

In early 2000, several cats were brought to a companion animal veterinary clinic (clinic C) with diarrhea; no stool specimens were collected. Three days after their cat was treated for vomiting and diarrhea, 2 children became ill with diarrhea. Six days after treating an ill cat, a clinic C employee also became ill with diarrhea. The duration of illness for the 3 ill persons was 5–7 days, and all 3 persons sought medical care. One child was hospitalized for 2 nights, but salmonellae were never cultured from this child's stool. Stool specimens obtained from the ill clinic employee, an asymptomatic clinic employee, and the other child yielded salmonellae, which were identified as *S*. Typhimurium at the Washington State Department of Public Health Laboratory. Clinic C was the only known association between the 3 ill persons.

During this period, stool specimens submitted from 1 dog and 3 cats with diarrhea yielded salmonellae; isolates were serotyped as *S*. Typhimurium by NVSL. Two days before diarrhea onset, the 4 animals had been treated as outpatients at clinic C for unrelated reasons; 3 of the 4 received amoxicillin before diarrhea onset.

The FDIU performed an investigation in clinic C, including bacterial cultures of stool specimens from additional animals (n = 96) and environmental samples from clinic C and employee and client homes (n = 66). *S*. Typhimurium was isolated from 1 cat and 2 dogs (1 asymptomatic). The cat exhibited anorexia and dehydration; a stool specimen was collected. This cat had not been seen at clinic C during the outbreak, but several of its housemates had been seen at clinic C for conditions other than diarrhea during the outbreak. One of the 2 dogs boarded at the clinic during the outbreak and had a single episode of bloody diarrhea; its asymptomatic housemate had not been seen by clinic C.

None of the environmental samples collected from clinic C yielded salmonellae. The outbreak strain of *S*. Typhimurium was identified in the contents of the home vacuum cleaner bags from 2 clinic C employees and the asymptomatic owner of a culture-confirmed cat (24). The *S*. Typhimurium isolates from the 2 ill persons, 7 animals, and the 3 vacuum bags were all demonstrated at CDC to be *S*. Typhimurium R-type ACSSuT DT104. These isolates were additionally indistinguishable by PFGE at the FDIU Laboratory, Pullman, Washington (Figure 3) by using standard protocols (25) and were indistinguishable from those of the clinic B outbreak.

## Discussion

Four outbreaks of multidrug-resistant *S*. Typhimurium associated with companion animal veterinary clinics or shelter facilities occurred in the United States in late 1999 and early 2000. In each facility, employees, clients, or both, became infected after animal illness. An outbreak in an Idaho clinic was caused by multidrug-resistant *S*. Typhimurium R-type ACKSSuT with 2 isolates demonstrating additional resistance to ceftriaxone, an antimicrobial agent commonly used to treat children with severe *Salmonella* infections. Outbreaks in 2 Washington clinics and a Minnesota animal shelter were caused by multidrug-resistant *S*. Typhimurium R-type ACSSuT DT104.

In 1999, *S*. Typhimurium R-types ACSSuT, AKSSuT, and ACKSSuT were the most prevalent multidrug-resistant phenotypes among *Salmonella* isolates in the United States. Twenty-one percent of all *Salmonella* isolates of human origin tested at the National Antimicrobial Resistance Monitoring System (NARMS) in 1999 were multidrug-resistant; 6% were *S*. Typhimurium R-type ACSSuT, 2% were R-type AKSSuT, and 1% were R-type ACKSSuT (11).

In each outbreak discussed, the veterinary facility or animal shelter was the only common exposure for infected persons, which demonstrated that infected animals brought to companion animal veterinary clinics and animal shelters can be foci for nosocomial transmission to other animals and for zoonotic transmission to humans. These outbreaks illustrate 1) the hazards of occupational zoonotic transmission of *Salmonella* spp. from ill animals to clinic employees, 2) the hazards of zoonotic transmission of *Salmonella* spp. to clients/pet owners, 3) the risk for nosocomial transmission of *Salmonella* spp. between animals within veterinary facilities and animal shelters, and 4) the potential for environmental contamination to serve as an ongoing source of infection.

The use of antimicrobial agents prescribed by veterinarians may contribute to increased transmission of multidrug-resistant *Salmonella* spp. between animals by lowering the infectious dose required for infection to occur or by increasing the duration of illness when an infected animal is treated with an ineffective drug (12,26,27). The risk for *Salmonella* transmission between animals in veterinary facilities is likely increased by the presence of animals with increased susceptibility to multidrug-resistant *Salmonella* infection due to treatment with antimicrobial agents for other conditions. Fluoroquinolone antimicrobial therapy did not eliminate fecal shedding of susceptible strains of salmonellae in 3 cats and 1 person from whom follow-up cultures were obtained. This finding is similar to other results in humans, which demonstrate that fluoroquinolone treatment is associated with longer duration of carriage (28).

Although person-to-person transmission of nontyphoidal *Salmonella* spp. is rare in the United States, this mode of transmission appears likely in the Minnesota outbreak in which an ill child apparently infected classmates in a daycare center. Several instances of probable secondary transmission to animals within client households after apparent primary nosocomial infection were demonstrated during these outbreaks. The isolation of *Salmonella* spp. from client-owned vacuum cleaner bags illustrated the potential for such secondary transmission. Additional isolation of the outbreak strain from environmental surfaces in the Washington clinic B investigation reinforces the findings of previous studies, which demonstrated the potential to transmit salmonellae through environmental contact (29,30).

Veterinarians should expect, at least occasionally, to evaluate animals infected with *Salmonella* spp. Following these outbreaks, recommendations for infection prevention and control were formulated to help prevent future outbreaks of salmonellosis in association with companion animal facilities (31). Recommendations include wearing gloves while cleaning cages and treating animals, then immediately removing the gloves and washing hands when the task is completed. No eating or drinking should be allowed in animal treatment and holding areas, and feces-contaminated areas should be immediately cleaned and disinfected. Clear warnings of the risk for transmission of *Salmonella* spp. should be given when pets with probable salmonellosis are encountered. Veterinarians should consider culturing the stools of animals with diarrhea and should be aware of the increased risk for infection with multidrug-resistant salmonellae in animals who are given antimicrobial drugs for other conditions. Because use of antimicrobial agents contributes to increasing resistance and facilitates transmission of multidrug-resistant salmonellae, promoting guidelines aimed at improving appropriate use of antimicrobial agents may help prevent transmission of multidrug-resistant *Salmonella* infections in veterinary facilities (31).

Although recommendations have been formulated and disseminated (31), outbreaks of multidrug-resistant *Salmonella* spp. occurred in an animal shelter in Idaho in 2003–2004 (18) and in New York in 2003 (19). Continued outbreaks suggest that more outreach and education regarding the potential for nosocomial and zoonotic outbreaks such as these should be directed to the veterinary community.

Additionally, states should consider integrating human and veterinary surveillance systems and educating the veterinary community on their public health role. In our report, the outbreak in 1 state may have been undetected without the routine comparison of human and veterinary laboratory data. Considering recent outbreaks of zoonotic diseases such as West Nile virus and monkeypox infections, the continued threat of avian influenza, and the number of agents of bioterrorism that are zoonotic in nature, the integration of human and veterinary surveillance systems is of utmost importance in our public health infrastructure.
